# Effect of *Pichia kudriavzevii* on Protein‐Related Techno‐Functional Properties of Fermented Sorghum Slurry to Manufacture Ting

**DOI:** 10.1155/ijfo/6574451

**Published:** 2026-11-07

**Authors:** Jennifer Selasie King, Andries Masenge, Mohammad Naushad Emmambux, Ngwekazi Nwabisa Mehlomakulu

**Affiliations:** ^1^ Department of Consumer and Food Sciences Old Agriculture Building University of Pretoria Hatfield South Africa up.ac.za; ^2^ Department of Statistics University of Pretoria Hatfield South Africa up.ac.za

**Keywords:** fermentation, *Pichia kudriavzevii*, protein, sorghum, ting

## Abstract

The yeast *Pichia kudriavzevii* was used as an adjunct starter culture to determine protein‐related functional properties of sorghum in the making of ting. The slurry was inoculated with the yeast to reach 3 Log_10_ CFU/g (IN3), 5 Log_10_ CFU/g (IN5), and 7 Log_10_ CFU/g (IN7), whereas spontaneously fermented sorghum (SP) served as the control. In IN5 and IN7, the coliform population was below the limit of detection from 24 h onward, whereas in SP the population was undetectable from 60 h, highlighting the antimicrobial properties of the yeast inoculum. Yeast inoculation did not negatively affect the lactic acid bacteria (LAB) population, which remained above 7 Log_10_ CFU/g from 12 h onward across all treatments. Yeast inoculated treatments (IN3 and IN5) had significantly higher (*p* < 0.05) water solubility index (WSI), nitrogen solubility index (NSI) and free amino nitrogen (FAN) from 48 h compared with SP and IN7. Protein content remained unchanged in all the treatments. In vitro protein digestibility (IVPD) improved (> 77%) in all the treatments at 48 h. At 72 h, only the SP IVPD showed a significant difference from 48 h. Starch granules in IN3, IN5, and IN7 treatments showed large pitting from 24 h to 72 h compared with SP. Overall, IN3 and IN5 hastened the fermentation process, provided a slurry with changes in techno‐functional properties, nondetectable coliforms, and increased IVPD.

## 1. Introduction

Sorghum porridges and gruels such as ting, gowé, togwa, motoho, and ogi are traditionally produced through spontaneous lactic acid fermentation. A diverse microbial population dominates the fermenting slurry and finished product. Lactic acid bacteria (LAB) species such as *Lactiplantibacillus plantarum*, *Limosilactobacillus fermentum*, *Pediococcus pentosaceus*, *Weissella confusa*, and *Lacticaseibacillus paracasei* dominate the fermentation [1–5]. A yeast population comprising *Saccharomyces cerevisiae*, *Debaryomyces hansenii*, *Geotrichum silvicola*, *Rhodotorula mucilaginosa*, *Kluyveromyces marxianus*, *Wickerhamomyces anomalus*, and *Pichia kudriavzevii* has been reported in slurries and their respective products [1, 2, 6–8]. The environment, equipment, and substrate materials used in spontaneous fermentations are reported as contributors to the microbiota in fermented gruels and porridges [9–11].

LAB and yeasts exist in a mutualistic interaction during the fermentation of cereals [12] as observed by the growth of *Lp. plantarum* when coinoculated with either *Candida krusei* or *S. cerevisiae* in maize [13]. The activity of the amylolytic LAB species degrades starch [14, 15] into available sugars for the fermentative LAB [2]. Species such as *Lp. plantarum*, *Lb. fermentum*, *Lactococcus lactis*, and *W. confusa* drive the acidification of the matrix by converting the sugar to lactic, succinic and citric acid during fermentation [5, 16]. This acidic environment is favorable for yeast growth, which produces amino acids [17] such as lysine and methionine, which are growth factors for LAB [18, 19].

The microbial interactions modify the matrix of cereals by hydrolyzing starch and proteins, thereby affecting the protein and tannin content of the substrate, in vitro antioxidant activity, in vitro protein digestibility, flavor profile, and mineral bioaccessibility [18–28]. Yeasts and LAB secrete extracellular enzymes such as phytase [29, 30], amylase [31], and protease [32] into the matrix during fermentation.

Fermentation of dehulled maize grains with *S. cerevisiae* and *Levilactobacillus brevis* co‐inoculum increased the essential amino acids lysine and methionine. Lysine was reported between 1.4 and 1.8 mg/g from Day 4 to 10. Although methionine was between 0.3 and 0.4 mg/g from Day 3 to 5 [18]. These amino acids are often limited in cereal‐based diets; thus, the coinoculated fermentation is favorable. Coinoculation of *S. cerevisiae* and *Lactobacillus delbrueckii* subsp. *bulgaricus* yielded a 76.59% protein content increase in malted sorghum compared to 55% in unmalted sorghum. In malted sorghum, the protein content was 68.4% for *S. cerevisiae* inoculation, whereas for *L. delbrueckii* subsp. *bulgaricus*, the protein content was 34.98%. In unmalted sorghum, the protein content was 58.2% for *S. cerevisiae* and 39.36% for *L. delbrueckii* subsp. *bulgaricus* axenic inoculation [28]. Whereas inoculation with *Lb. plantarum* increased protein digestibility by 92% compared with 47% in spontaneous fermentation [33]. A LAB consortium of *Lb. fermentum*, *Lb. brevis*, *Companilactobacillus nantensis*, *Pediococcus acidilactici* and *Lb. plantarum* led to an IVPD increase from 63.68% to 84.28%, compared with a 79.89% increase from spontaneous fermented sorghum [24]. Although tannin content decreased in ting prepared from either high‐tannin and low‐tannin whole‐grain sorghum fermented with either *Lb. fermentum* strains FUA 3165 and 3321 axenically or as a co‐inoculum. Ting fermented from high‐tannin sorghum yielded a tannin content of 2.83 mg CE/g (strain FUA 3165), 2.61 mg CE/g (strain FUA 3321), and 3.01 mg CE/g (coinoculated strains) compared with spontaneous fermented ting at 14.11 mg CE/g. Whereas fermentation of low‐tannin sorghum resulted in a tannin content of 0.52 mg CE/g (strain FUA 3165), 0.41 mg CE/g (strain FUA 3321), and 0.63 mg CE/g (coinoculated strains) compared with spontaneous fermented ting at 5.36 mg CE/g [22].

The yeast *P. kudriavzevii* has been isolated in fermented sorghum porridges and gruels [7, 8, 34, 35] because of its tolerance to low pH and oxidative stress during fermentation [36]. *P. kudriavzevii* was reported to contribute a 1.1% increase in leucine, 5.9% to 8.7% increase in phenylalanine, and 11.2% in lysine when inoculated in maize‐based mawè [37]. Whereas when coinoculated with *Lb. fermentum* for fermentation of pearl millet gruel, a notable increase (between 8 *μ*g/100 g and 10 *μ*g/100 g) in folate content was observed within 4 h of fermentation [38]. In a study conducted by Ogunremi et al. [34], *P. kudriavzevii* improved flavor‐active compounds and antioxidant activity of ogi. The authors attributed *P. kudriavzevii*′s ability to enhance flavor properties by utilizing sugars and amino acids in the matrix to produce organic acids, benzyl alcohol, octadecane, tetradecanal and octadecanal.

Investigating the contribution of yeasts in sorghum fermentation could provide insights into optimizing fermentation processes, the development of yeast starter cultures for the production of shelf‐stable, nutritionally adequate and functional indigenous foods. Therefore, the objectives of this study were to determine the effect of *P. kudriavzevii* as an adjunct starter culture on the indigenous LAB population, protein‐related functional properties, and fermentation duration during the making of ting. The aim of these was to improve the protein quality of sorghum ting.

## 2. Materials and Methods

### 2.1. Spontaneous Fermentation of Ting

Coarse sorghum meal (for making ting) was procured from a supermarket in Pretoria, South Africa. The initial particle size (> 1.4 mm) of the meal was further reduced by milling 500 g of sorghum meal for 30 s in a blender (Russel Hobbs) with stirring at 10 s intervals. The final particle size was determined with British standard laboratory test sieves Series ISO 3310:2000 (nominal aperture size) and were 8% > 1.7 mm, 32% between 1.4–1.7 mm, 44% between 1.0 and 1.4 mm, and 16% < 1 mm. A slurry was prepared by mixing coarse sorghum meal with sterile distilled water at 50°C at a 1:1 (w/v) ratio in clean, sanitary containers. The slurry was incubated at 30°C for 72 h and sampled aseptically at 0, 12, 24, 36, 48, 60 and 72 h for analysis described below.

### 2.2. Fermentation of Ting With Yeast Adjunct Starter Culture

*P. kudriavzevii* strain CBS 2050, purchased from Westerdjik Fungal Biodiversity Institute, Netherlands, in its lyophilized state, was revived following the manufacturer′s instructions. The strain was preserved in yeast peptone dextrose (YPD) broth (Merck, Darmstadt, Germany) in 50% sterile glycerol in cryotubes at −80°C.

The inoculum for fermentation was prepared following the procedure outlined by [39] with modifications. The frozen vials were thawed at room temperature, streaked with a sterile loop onto YPD agar (Merck, Darmstadt, Germany) and incubated at 30°C for 72 h. A single, pure colony was isolated and inoculated into 10 mL YPD broth, then incubated at 30°C for 24 h to prepare the preculture. The inoculum was prepared by inoculating 1 mL of the 24 h preculture into 100 mL of YPD broth in a conical flask and incubated at 30°C in a shaking water bath at 200 rpm from 18 h to 20 h. The 100 mL culture underwent centrifugation for 10 min at 7670 × *g* at 4°C. The pellet was washed thrice in 0.1% sterile NaCl then resuspended in 10 mL of 0.1% sterile NaCl and vortex mixed. Cell concentration of the resuspended culture was determined by serial dilution in 0.1% peptone‐buffered water and plating on YPD agar (Merck, Darmstadt, Germany). The agar plates were incubated at 30°C for 120 h and agar plates with counts ranging from 30 to 300 colony‐forming units (CFU) were enumerated. The cell concentration was 8 Log_10_ CFU/mL.

The slurry for the inoculated fermented sorghum was prepared by adding coarse sorghum meal to sterile distilled water in a 1:1 ratio (w/v). The slurry was inoculated with the yeast inoculum to attain a final cell concentration of 3 Log_10_ CFU/g (IN3), 5 Log_10_ CFU/g (IN5), and 7 Log_10_ CFU/g (IN7) in the slurry. Fermentation was carried out at 30°C for 72 h. The fermenting slurries were sampled aseptically at 0, 12, 24, 36, 48, 60, and 72 h for analysis described below.

### 2.3. Determination of pH and Lactic Acid Percentage

The pH and lactic acid % of the fermenting slurry were measured at 0, 12, 24, 36, 48, 60, and 72 h to monitor the fermentation. The pH of each sample was measured in triplicate using a calibrated pH meter (Hanna Instruments, Woonsocket, Rhode Island, United States), with standard buffer solutions of pH 4.0 and 7.0. Lactic acid concentration was done using a titration method. A 2 g mass of the fermented sorghum slurry was diluted with 20 mL of distilled water. This diluted slurry was titrated against 0.1 N NaOH (Sigma–Aldrich, Kempton Park, Gauteng Province, South Africa) using three drops of phenolphthalein as an indicator until the mixture turned pale pink. Total lactic acid was calculated using Formula (1).

(1) Lactic acid%=N×ME of lactic acidWeight of sample g×100

*N* = Normality of NaOH

*M* *E* = Milli − equivalent of lactic acid (0.09008)

*V* = NaOH in mL to reach the titration endpoint

### 2.4. Microbiological Enumeration

To monitor the microbial population during fermentation, samples were analyzed at 0, 12, 24, 36, 48, 60, and 72 h. Each of the fermenting slurries was sampled by weighing 10 g, which was diluted with 90 mL of sterile peptone‐buffered water and homogenized for 1 min using a Colworth Stomacher 400 Laboratory blender (Art Medical Equipment Pty Ltd, Johannesburg, South Africa). The mixture was serially diluted 10‐fold in peptone‐buffered water, and 0.1 mL of the appropriate dilutions was enumerated by surface plating on different agar media, as outlined below.

Yeasts were enumerated on Malt Yeast Glucose Peptone with copper agar (Sigma–Aldrich, St. Louis, United States) supplemented with 50 mg/L of chloramphenicol (Sigma–Aldrich, St. Louis, United States) and 25 mg/L of tetracycline (Sigma–Aldrich). De Man–Rogosa and Sharpe (MRS) agar (Merck, Darmstadt, Germany) was used to enumerate total LAB. The total aerobic count was enumerated on plate count agar (Merck, Darmstadt, Germany) and Violet Red Bile Glucose (VRBG) agar (Sigma‐Aldrich, St. Louis, United States) was used to enumerate coliforms.

All the total yeast and aerobic counts agar plates were incubated at 30°C for 120 h, whereas the total LAB and coliforms agar plates were incubated at 37°C for 24 h. After incubation, agar plates with 30 to 300 CFU” were enumerated and reported as Log_10_ CFU/g. The mean value and standard deviation were calculated for each sample.

### 2.5. Determination of Water Solubility Index (WSI) and Water Absorption Capacity (WAC)

To monitor the effect of the fermentation on WSI and WAC, samples were analyzed at 0, 12, 24, 36, 48, 60, and 72 h. The WSI was determined following the procedure outlined by [40] with a few modifications. A conical centrifuge tube was filled with 30 mL of distilled water, to which 2.5 g of fermented slurry sample was added, and the mixture was shaken thoroughly. The mixture was kept warm in a shaking water bath at 30°C for 30 min and vortex‐mixed at 5 min intervals. The mixture was cooled to room temperature, then centrifuged at 3170 × *g* for 10 min at 25°C. The supernatant was decanted into a preweighed evaporating tin whereas the pellet was weighed separately. Subsequently, the supernatant was evaporated overnight at 100°C in an oven to recover dry solids. The weight of the dry solids was measured. The WSI and WAC were calculated using the Formula (2) and (3), respectively.

(2) WSI %=weight of dry solids in supernatantoriginal weight of sample×100

(3) WACg/g=weight of pellet−original weight of sampleoriginal weight of sample

### 2.6. Determination of Nitrogen Solubility Index (NSI)

To monitor the effect of the fermentation on NSI, samples were analyzed at 0, 12, 24, 36, 48, 60, and 72 h. The evaluation of “NSI” was carried out according to the method described in AACC Method 46‐23 [41]. A total of 1 g of each fermented slurry was mixed with 20 mL of 0.1 M NaCl solution (pH 7) and agitated for an hour at 30°C in a shaking water bath. The suspension was centrifuged at 9160 × *g* for 15 min at 30°C. The resulting supernatant was filtered using a Whatman No. 4 filter paper. The residue obtained postfiltration was rinsed twice with 10 mL of 0.1 M NaCl solution (pH 7). The filtrate was then frozen at −20°C and subsequently freeze‐dried for 4 days. The nitrogen content of the freeze‐dried samples was determined using a “Dumatherm (DT, Gerhardt Konigswinter, Germany). NSI was calculated on a dry basis using Formula (4).

(4) NSI%=Nitrogen content of freeze−dried sampleTotal nitrogen content of fermented sorghum×100

### 2.7. Determination of Free Amino Nitrogen (FAN)

To monitor the effect of the fermentation on FAN, samples were analyzed at 0, 12, 24, 36, 48, 60, and 72 h. Using glycine as a standard, FAN was determined using the ninhydrin colorimetric assay, EBC Method 8.8.1” [42] stated by [43]. The results are presented as FAN (mg/100 g) sorghum on a dry‐weight basis.

### 2.8. Determination of Protein Content

To monitor the effect of the fermentation on protein, samples were analyzed at 0, 12, 24, 36, 48, 60, and 72 h. Protein content of all samples was analyzed using the Dumas method, as described by Shea and Watts [44], with the aid of the Dumatherm nitrogen combustion system (DT, Gerhardts, Konigswinter, Germany). The total protein content was calculated using the factor of *N* (Nitrogen percentage) × 6.25.

### 2.9. In Vitro Protein Digestibility of Sorghum Ting

Based on the pH, lactic acid %, and functional properties, IVPD was measured at 0, 48, and 72 h to correspond to a reference time point (0 h) and to align with the end of fermentation (48 h and 72 h) of artisanal‐produced ting. In vitro protein digestibility of the fermented slurries at 0, 48, and 72 h was determined using a multienzyme technique [45]. The multienzyme solution used for digestion contained 16 mg/mL of trypsin from bovine pancreas (14 600 U/mg, Sigma–Aldrich, St. Louis, United States), 3.1 mg/mL of *α*‐chymotrypsin from bovine pancreas (40 U/mg, Sigma–Aldrich, St. Louis, United States) and 1.3 mg/mL of protease from *Streptomyces griseus* (≥ 3.5 U/mg solid, Sigma–Aldrich, St. Louis, United States). A total of 5 mL of the chilled enzyme solution was mixed with each fermented slurry suspension (protein concentration = 6.25 mg/mL, pH adjusted to 8.0 with 0.1 M NaOH). The mixture was incubated at 37°C in a shaking water bath and the decrease in pH was recorded after 10 min. The linear regression equation was calculated using Equation (5).

(5) y=210.4618.10−x

where *y* = *in* *v* *i* *t* *r* *o* *p* *r* *o* *t* *e* *i* *n* *d* *i* *g* *e* *s* *t* *i* *b* *i* *l* *i* *t* *y* (%) and *x* = *p* *H* *of* *the* *s* *a* *m* *p* *l* *e* *s* *u* *s* *p* *e* *n* *s* *i* *o* *n* *a* *f* *t* *e* *r* 10 min *i* *n* *c* *u* *b* *a* *t* *i* *o* *n*.

### 2.10. Morphological Changes of the Fermenting Sorghum Slurry

Fermented slurry samples collected at 0, 24, 48, and 72 h were observed under a scanning electron microscope for protein and starch morphological changes using the methods of [21, 46] after freeze‐drying. An aluminium stub was prepared with an adhesive carbon tape to which carbon was coated using the Quorum Q150TE instrument (Quorum Technologies, Sussex, United Kingdom). The freeze‐dried samples were then fixed onto the stub and examined using a Zeiss 540 Ultra Scanning Electron Microscope (Zeiss 540 Ultra PLUS FEG; Zeiss Microscopy, Göttingen, Germany) in vacuum at a 10 kV acceleration voltage. All images were captured at magnifications of 5x and 10x.

## 3. Statistical Analysis

Each experiment was conducted in triplicate. All collected data underwent statistical analysis using a single‐factor analysis of variance (ANOVA) and Duncan′s multiple range test for mean separation; significant differences were indicated at *p* ≤ 0.05. Statistics were analyzed using IBM SPSS software Version 28.0.0.0 (IBM Corp., Armonk, New York, United States).

## 4. Results and Discussion

### 4.1. Physicochemical Analysis of Fermenting Sorghum Slurry

All fermenting ting slurries exhibited a decrease in pH and an increase in lactic acid concentration (Figure 1a,b). The SP and IN3 treatments showed a gradual decrease in pH from 6.3 to 5.8 and 6.0 at 12 h (Figure 1a), corresponding to lactic acid concentrations of 0.24% and 0.17%, respectively (Figure 1b). An increase in the inoculum concentration for IN5 and IN7 samples resulted in pH values of 5.3 and 4.9 and lactic acid contents of 0.20% and 0.34%, respectively, at the same time point. These findings are similar to those reported by [37] in fermented maize dough inoculated with strains of *P. kudriavzevii* at 6 Log_10_ CFU/g, where pH decreased between 4.5 and 6.0 after 12 h of fermentation from pH 6.2. The significant (*p* ≤ 0.05) decrease in pH observed in IN5 and IN7 at 12 h aligns with a sharp rise in LAB population (Figure 2a) of 7.71 and 9.15 Log_10_ CFU/g, respectively.

**Figure 1:**
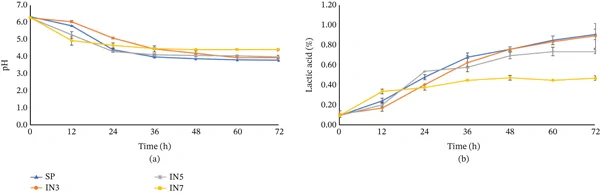
Effects of spontaneously and *Pichia kudriavzevii* inoculated sorghum on (a) pH and (b) lactic acid during fermentation for ting production.

**Figure 2:**
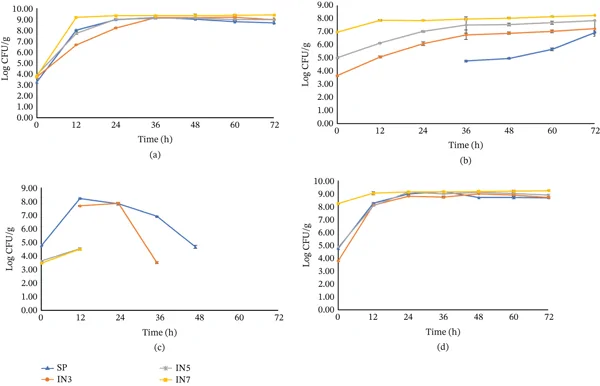
Effects of spontaneously and *Pichia kudriavzevii* inoculated sorghum on (a) LAB population, (b) yeast population, (c) coliform population, and (d) total aerobic count during fermentation for ting production. IN3, inoculated sorghum at 3 Log_10_ CFU/g; IN5, inoculated sorghum at 5 Log_10_ CFU/g; IN7, inoculated sorghum at 7 Log_10_ CFU/g; SP, spontaneously fermented sorghum.

At the end of the fermentation period (Figure 1a), SP had a pH of 3.8 and lactic acid was at 0.92%, which was significantly higher (*p* ≤ 0.05) than the lactic acid for IN5 (0.74%) and IN7 (0.47%) at pH 4.0 and 4.42, respectively. The spontaneous fermentation of ting is reported to have a final pH between 3.5 and 4.0 because of the dominance of LAB population [47] which is in agreement with the results of this study.

In the yeast treatments—IN3, IN5, and IN7 there was a slow and nonsignificant (*p* ≥ 0.05) change in pH and lactic acid percentage from 24 h to 72 h. This is attributed to *P. kudriavzevii* possessing CYB2Ap genes linked to lactic acid assimilation thus relieving acid stress [19, 48].

### 4.2. Microbial Population During Sorghum Fermentation

The yeast‐inoculated treatments exhibited an initial LAB population between 3.70 and 3.93 Log_10_ CFU/g, whereas the SP treatment had a LAB population of 3.20 Log_10_ CFU/g. All treatments reached a LAB population between 8 and 9 Log_10_ CFU/g at the end of fermentation. These results are similar to the LAB population reported by [47], which comprised of *Lp. plantarum*, *Lacticaseibacillus casei*, *Schleiferilactobacillus harbinensis*, *Lb. fermentum, Lentilactobacillus parabuchneri*, *Limosilactobacillus reuteri*, and *Loigolactobacillus coryniformis.*

The yeast‐inoculated treatments (IN3, IN5, and IN7) exhibited a steady LAB population exceeding 7 Log_10_ CFU/g from 24 h until the end of fermentation. The inoculation of yeasts did not inhibit the inherent LAB population in these treatments. Yeasts can coexist with LAB by assimilating lactic acid during fermentation [49] and providing vitamins and nitrogen sources, such as L‐amino acids, to LAB for growth [50].

The increase in LAB during the early stages of fermentation did not inhibit the growth of *P. kudriavzevii* in the IN3, IN5, and IN7 treatments (Figure 2b). This confirmed the mutualistic interaction between these microbial groups [51] and is consistent with other reported findings [13, 37] in cereal fermentation. Whereas in the SP, the yeast population remained below the limit of detection for the first 24 h (Figure 2b) and this is attributed to the low (undetectable) yeast population in the sorghum slurry prior to active fermentation. However, at 36 h the yeast population increases to a detectable population and this growth is supported by the nutrient content provided by amylolactic LAB which breakdown starch to fermentable substrates such as glucose. Furthermore, LAB produces lactic acid from glucose [16], which creates an acidic environment favorable for yeast growth [37, 52]. Overall, the interaction between these microbial groups was also observed in the total aerobic count population (Figure 2d). SP′s initial Enterobacteriaceae population was higher (4.73 Log_10_ CFU/g) than IN3, IN5, and IN7 (between 3 and 4 Log_10_ CFU/g). This population decreased variability across all treatments to below the limit of detection at 60 h. In IN 5 and IN7, the population was undetectable from 24 h, IN 3 from 48 h, and SP from 60 h (Figure 2c). Similar findings were observed during the fermentation of kenkey, a thick maize porridge, in which low levels of gram‐negative bacteria, including Enterobacteriaceae, were reported at a pH of 3.96 [53]. For the yeast treatments IN3, IN5, and IN7, the below limit of detection of coliforms correlated to pH levels of (4.20, 4.32, and 4.67, respectively) than SP at pH 3.83. In the yeast treatments, the coliform decline is attributed to antimicrobial compounds produced by *P. kudriavzevii* (alcohols and carbon dioxide) and LAB (lactic acid and aldehydes), which inhibit coliforms such as *Pseudomonas aeruginosa*, *Klebsiella pneumoniae*, and *Escherichia coli* [54]*. P. kudriavzevii* was reported to exhibit antagonistic activity against various pathogens, for example, *Enterococcus faecalis*, *Micrococcus luteus*, *K. pneumoniae*, *Salmonella typhi*, *P. aeruginosa*, and *Vibrio cholerae* by production of organic acids and secondary metabolites present in the supernatant [55].

### 4.3. Protein Content Fermented Sorghum Slurry and IVPD of Fermented and Cooked Sorghum Ting

Fermentation did not significantly (*p* ≥ 0.05) alter protein content across treatments (SP, IN3, IN5, and IN7) (Table 1). During fermentation (e.g. spontaneous), protein is released from the grain matrix through proteolysis to different protein forms (e.g. soluble nitrogen, amino acid, and peptides). Additionally, other biochemical reactions occur, such as the utilization of amino acids and peptides by yeast and LAB via the Ehrlich Neubauer pathway. This involves transamination and decarboxylation, leading to the production of aroma compounds such as benzenic aldehydes, alcohols, esters, and acids [56]. Protein is determined by nitrogen content in the current study, multiplied by the conversion factor to obtain the protein value. Thus, even though there are biochemical changes in the protein, the nitrogen content in the system did not change.

**Table 1:** Effects of spontaneous and *Pichia kudriavzevii* inoculated fermentation on the protein content of sorghum ting.

Time (h)	0	12	24	36	48	60	72
Protein content (%)							
SP	10.23 ± 0.14^Aa^	10.80 ± 0.50^ABa^	11.0 ± 0.07^BCa^	11.6 ± 0.36^Ca^	11.6 ± 0.31^Ca^	11.4 ± 0.16^Ca^	11.3 ± 0.43^BCa^
IN3	10.23 ± 0.14^Aa^	10.45 ± 0.42^ABa^	10.69 ± 0.41^ABCa^	11.06 ± 0.41^BCab^	10.91 ± 0.26^BCb^	10.75 ± 0.07^ABCb^	11.15 ± 0.36^Ca^
IN5	10.23 ± 0.14^Aa^	10.86 ± 0.12^BCa^	10.96 ± 0.20^Ca^	10.82 ± 0.13^BCbc^	10.58 ± 0.17^Bb^	10.60 ± 0.14^Bbc^	10.72 ± 0.16^BCa^
IN7	10.23 ± 0.14^Aa^	10.64 ± 0.56^Aa^	10.17 ± 0.16^Ab^	10.30 ± 0.16^Ac^	10.68 ± 0.24^Ab^	10.37 ± 0.32^Ac^	11.42 ± 0.40^Ba^

*Note:* Results reported as mean ± SD (*n* = 3 throughout the fermentation period). Mean values with different uppercase superscripts are significantly different (*p* ≤ 0.05) across the time points for each treatment. Mean values with different lowercase superscripts are significantly different (*p* ≤ 0.05) within the treatments for each time point.

Abbreviations: IN3, inoculated sorghum at 3 Log_10_ CFU/g; IN5, inoculated sorghum at 5 Log_10_ CFU/g; IN7, inoculated sorghum at 7 Log_10_ CFU/g; SP, spontaneously fermented sorghum.

At the household level, ting is cooked after 48 h or 72 h of fermentation when the desired sourness is reached. In the current study, the pH ranged between 3.5 to 4.5 in the fermentation period 48 h to 72 h. Thus, the IVPD time points studied corresponded with those in the study by Kewuyemi et al. [57]. The treatments IN3, IN5, and IN7 achieved higher IVPD at 79.90%, 79.54%, and 78.45%, respectively, at 48 h than the SP treatment (77.73%). At 72 h, the yeast‐inoculated treatments were significantly higher (*p* ≤ 0.05) than SP. However, the IN3, IN5, and IN7 treatments were not statistically significant (*p* ≥ 0.05) from each other (Table 2). Inoculation of *S. cerevisiae* in sorghum increased IVPD to between 70% to 90% when pepsin was used at 0.02% and 0.2% according to the AOAC method 971.09 [58]. In this study, increased IVPD correlated with increased NSI and FAN (Table 3), indicative of protein hydrolysis. This is attributed to the sorghum protein being broken down by endogenous proteinases [33] and to proteases released by yeasts or LAB, which result in the release of soluble nitrogen, peptides, and amino acids into the matrix [59]. *P. kudriavzevii* is reported to possess protease activity [60] and contributed to the availability of peptides and amino acids in the matrix [61]. Furthermore, yeast biomass could have contributed to the available protein in the matrix.

**Table 2:** Effects of spontaneous and *Pichia kudriavzevii* inoculated fermentation on the in vitro protein digestibility of sorghum ting.

Time (h)	0	48	72
IVPD (%)			
SP	75.49 ± 0.21^Aa^	77.73 ± 0.21^Ba^	78.27 ± 0.10^Ca^
IN3	75.49 ± 0.21^Aa^	79.90 ± 0.21^Bb^	79.42 ± 0.65^Bb^
IN5	75.49 ± 0.21^Aa^	79.54 ± 0.89^Bb^	79.11 ± 0.63^Bab^
IN7	75.49 ± 0.21^Aa^	78.45 ± 1.16^Bab^	78.63 ± 0.21^Bab^

*Note:* Results reported as mean ± SD (*n* = 3 throughout the fermentation period). Mean values with different uppercase superscripts are significantly different (*p* ≤ 0.05) across the time points for each treatment. Mean values with different lowercase superscripts are significantly different (*p* ≤ 0.05) within the treatments for each time point.

Abbreviations: IN3, inoculated sorghum at 3 Log_10_ CFU/g; IN5, inoculated sorghum at 5 Log_10_ CFU/g; IN7, inoculated sorghum at 7 Log_10_ CFU/g; SP, spontaneously fermented sorghum.

**Table 3:** Effects of spontaneous and *Pichia kudriavzevii* inoculated fermentation on the functional properties of sorghum ting.

Time (h)	0	12	24	36	48	60	72
Water solubility index (%)							
SP	2.39 ± 0.88^Aa^	2.87 ± 0.40^ABab^	2.95 ± 0.16^ABab^	3.00 ± 0.25^ABa^	2.99 ± 0.33^ABa^	3.31 ± 0.14^Ba^	3.38 ± 0.13^Bab^
IN3	2.39 ± 0.88^Aa^	3.60 ± 0.15^BCc^	2.72 ± 0.05^Ca^	2.80 ± 0.08^Ca^	3.46 ± 0.81^BCa^	3.62 ± 0.73^BCa^	3.85 ± 0.49^Cb^
IN5	2.39 ± 0.88^Aa^	3.08 ± 0.25^Bb^	3.10 ± 0.05^Bb^	2.99 ± 0.09^Ba^	3.04 ± 0.19^Ba^	3.32 ± 0.58^Ca^	3.47 ± 0.12^Cb^
IN7	2.70 ± 0.09^ABa^	2.50 ± 0.06^Aa^	2.79 ± 0.29^ABab^	2.83 ± 0.19^Aba^	2.93 ± 0.14^Ba^	2.92 ± 0.21^Ba^	2.89 ± 0.20^Ba^
Water absorption capacity (g/g)							
SP	1.50 ± 0.04^Aa^	1.57 ± 0.04^Ab^	1.72 ± 0.09^Aba^	1.72 ± 0.12^ABa^	1.82 ± 0.26^Ba^	1.50 ± 0.07^Aab^	1.52 ± 0.11^Aab^
IN3	1.46 ± 0.05^ABa^	1.40 ± 0.03^ABab^	1.30 ± 0.04^Ab^	1.41 ± 0.11^ABb^	1.39 ± 0.00^ABb^	1.47 ± 0.04^Ba^	1.47 ± 0.04^Ba^
IN5	1.49 ± 0.10^ABa^	1.24 ± 0.03^Da^	1.32 ± 0.11^CDb^	1.39 ± 0.07^BCb^	1.39 ± 0.05^BCb^	1.60 ± 0.07^Ab^	1.45 ± 0.02^BCa^
IN7	1.56 ± 0.09^Aa^	1.86 ± 0.19^Bc^	1.68 ± 0.05^Ab^	1.81 ± 0.09^Ba^	1.81 ± 0.07^Ba^	1.59 ± 0.06^Aab^	1.62 ± 0.02^Ab^
Nitrogen solubility index (%)							
SP	13.40 ± 0.53^Aa^	15.48 ± 2.74^Aa^	15.90 ± 0.84^Aa^	20.24 ± 1.45^Ba^	23.61 ± 1.27^Ca^	25.27 ± 0.85^CDb^	26.79 ± 1.26^Db^
IN3	13.40 ± 0.53^Aa^	25.07 ± 0.10^CDc^	21.28 ± 1.09^Bb^	22.50 ± 2.27^BCa^	27.86 ± 0.68^Db^	28.74 ± 2.88^Eb^	28.14 ± 1.94^DEb^
IN5	13.40 ± 0.53^Aa^	29.15 ± 0.80^BCd^	28.81 ± 0.23^Bd^	31.11 ± 0.70^BCDb^	32.52 ± 2.72^Dc^	32.56 ± 2.24^Dc^	32.65 ± 1.08^CDc^
IN7	13.40 ± 0.53^Aa^	19.56 ± 0.79^Bb^	23.50 ± 0.67^Dc^	22.49 ± 1.80^CDa^	23.46 ± 0.96^Da^	21.37 ± 0.45^BCa^	20.10 ± 1.35^Ba^
Free amino nitrogen (mg/100 g)							
SP	64 ± 1.53^Aa^	71 ± 2.69^Aa^	69 ± 4.98^Aa^	79 ± 4.54^Bab^	86 ± 3.29^BCa^	89 ± 6.91^Cb^	102 ± 3.72^Db^
IN3	69 ± 2.41^Ab^	83 ± 3.90^Bb^	76 ± 3.64^ABb^	77 ± 4.65^Ba^	95 ± 0.95^Cb^	97 ± 3.59^Cbc^	110 ± 4.66^Da^
IN5	69 ± 2.41^Ab^	74 ± 1.47^Ba^	85 ± 1.48^Cc^	89 ± 0.69^Db^	93 ± 0.63^Eab^	102 ± 2.24^Fc^	102 ± 2.34^Fab^
IN7	69 ± 2.41^Ab^	76 ± 2.94^ABa^	78 ± 3.34^ABb^	79 ± 8.36^ABab^	86 ± 6.36^Ba^	77 ± 6.97^ABa^	81 ± 9.75^Ac^

*Note:* Results reported as mean ± SD (*n* = 3 throughout the fermentation period). Mean values with different uppercase superscripts are significantly different (*p* ≤ 0.05) across the time points for each treatment. Mean values with different lowercase superscripts are significantly different (*p* ≤ 0.05) within the treatments for each time point.

Abbreviations: IN3, inoculated sorghum at 3 Log_10_ CFU/g; IN5, inoculated sorghum at 5 Log_10_ CFU/g; IN7, inoculated sorghum at 7 Log_10_ CFU/g; SP, spontaneously fermented sorghum.

## 5. Functional Properties of Fermenting Sorghum Slurry

### 5.1. WSI of Fermenting Sorghum Slurry

WSI measures the breakdown of starch and proteins, leading to the formation of soluble molecules [62]. In this study, all fermentation treatments increased in WSI, with IN3 and IN5 showing a significant (*p* ≤ 0.05) increase compared with SP and IN7 (Table 3). At 60 and 72 h, all treatments had WSI greater than 2.80%. This can be attributed to the release of intermediate fermentation metabolites into the matrix. The treatment with the higher yeast inoculum (IN7) had a lower WSI than the other treatments during fermentation. This is attributed to the rapid assimilation of the metabolites by the higher microbial population.

### 5.2. WAC of Fermenting Sorghum Slurry

WAC is a quantitative measure of starch granules and other polymers′ ability to absorb water [63]. SP and IN7 exhibited the highest WAC at 72 h (1.52 g/g and 1.62 g/g, respectively), whereas IN3 and IN5 showed lower values (1.47 g/g and 1.45 g/g, respectively (Table 3). Adiandri and Hidayah [63] found that sorghum flour fermented with a higher inoculum of *L. casei* led to a reduction in WAC from 4–12 h. An inoculum concentration of 0.15% decreased the WAC from 15% to 14.43% whereas a 0.20% inoculum concentration reduced WAC from 14.74% to 14.03%. The proteins released into the matrix contributed to the water‐holding capacity of these treatments through their hydrophilic side chains. A low WAC is desirable because it enables the preparation of porridges with reduced moisture content and increased energy/nutrient density [64].

### 5.3. NSI and FAN of Fermenting Sorghum Slurry

Protein breakdown is observed in the NSI and FAN (Table 3) during fermentation. In all treatments, NSI and FAN increased during fermentation, which indicated an increase in soluble nitrogen compounds. This is consistent with findings from Onyango et al. [65] and Elkhalifa et al. [66], who reported that fermentation enhanced NSI in a maize‐finger millet blend and fermented sorghum flour, respectively. This increase is attributed to the proteolytic activity of endogenous and microbial enzymes, which break down proteins into amino acids and peptides [67]. SP, IN3, and IN5 exhibited a notable increase in NSI from 13.40% (0 h) to between 23.61% and 32.65% from 48 to 72 h. For IN7, the highest recorded NSI was 23.46% at 48 h, decreasing to 20.10% at 72 h. This also resulted in a reduced FAN of 81 mg/100 g by 72 h compared with the rest of the treatments which were greater than 100 mg/100 g. Nitrogen is a substantial element to sustain yeast growth [68] thus in IN7, the released nitrogen from proteolysis could have been utilized by the high yeast population.

## 6. Morphological Changes in Fermented Sorghum Slurry

Unfermented sorghum meal exhibited large, polygonal, tightly packed starch granules with a rough surface (Figure 3). These starch granules were enclosed within a protein body, consistent with the raw sorghum microstructures reported by [25]. Fermentation of the meal led to pitting formation, which was more pronounced in the yeast‐inoculated treatments (IN3, IN5, and IN7) than in spontaneously fermented sorghum (SP) (Figure 4a). At 48 h, SP (Figure 4b) developed noticeable pitting, resembling that observed in the yeast treatments. This was also observed at 72 h on all treatments (Figure 4c). Pitting formation is attributed to activity of amylase and protease enzymes [25]. The presence of *P. kudriavzevii* in IN3, IN5, and IN7 accelerated starch hydrolysis, forming pitted granules early (24 h) in inoculated samples, while pitting in SP became evident only at 48 to 72 h which corresponded with the rise in the microbial population. Furthermore, the prevailing acidic conditions led to the solubilizing of the protein matrix surrounding kafirin, making starch granules more accessible to enzymatic hydrolysis.

**Figure 3:**
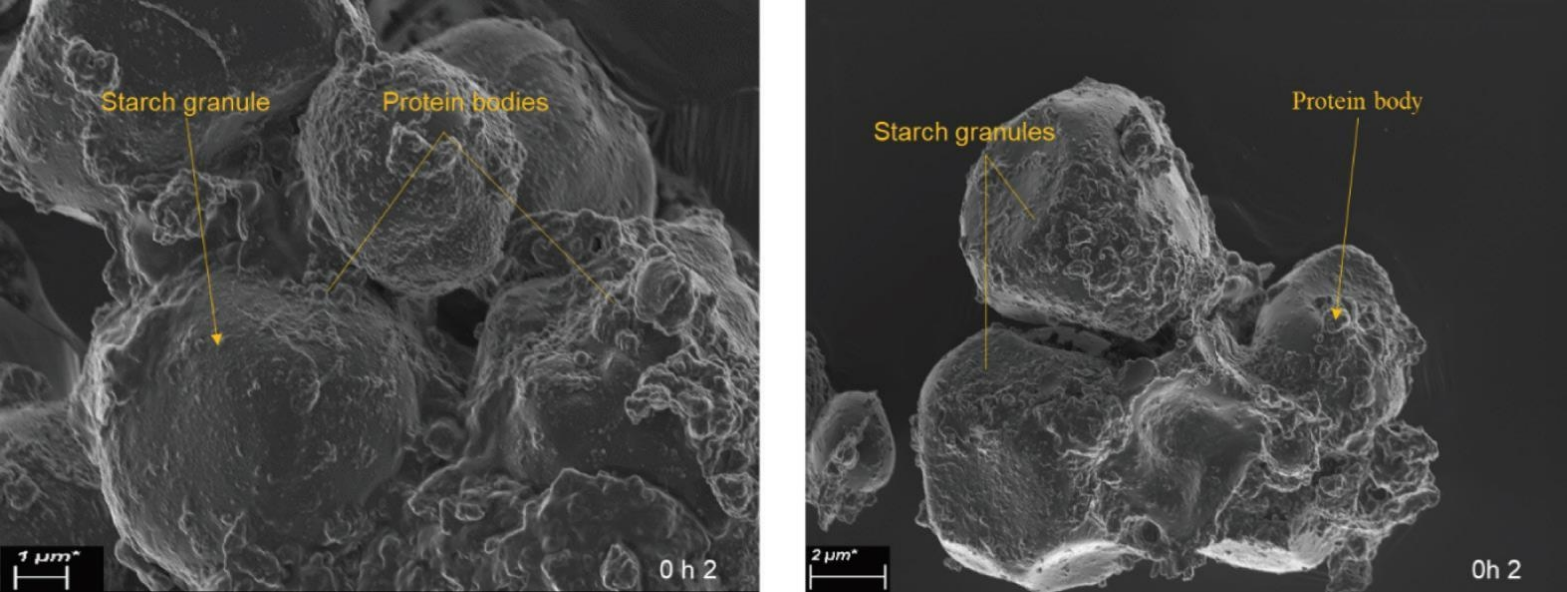
Scanning electron microscope images of unfermented sorghum meal.

**Figure 4:**
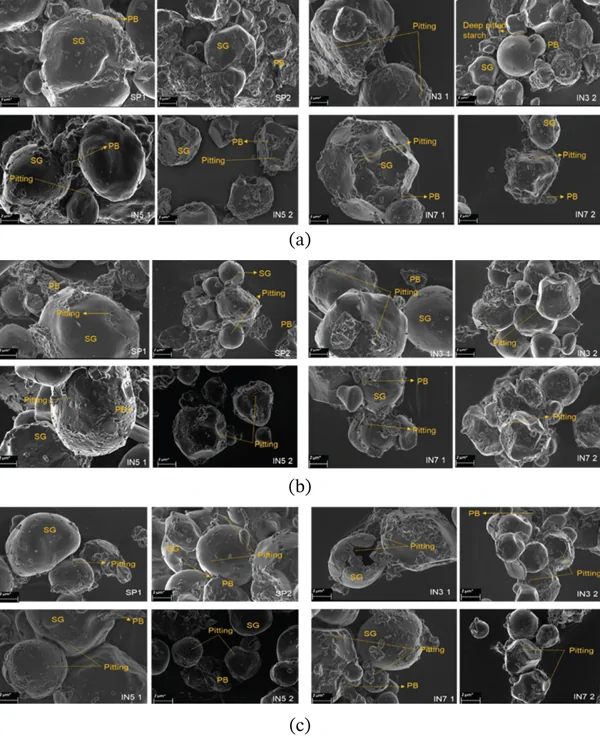
Scanning electron microscopy images of fermented sorghum for (a) 24 h, (b) 48 h, and (c) 72 h with inoculated *Pichia kudriavzevii.*1, x10,000 magnification; 2, x5000 magnification; IN3, inoculated at 3 Log_10_ CFU/g; IN5, inoculated at 5 Log_10_ CFU/g; IN7, inoculated at 7 Log_10_ CFU/g; PB, protein body; SG, starch granule; SP, spontaneously fermented.

## 7. Conclusions

This study has evaluated the use of *P. kudriavzevii* at different inoculum concentrations in a sorghum ting slurry. *P. kudriavzevii* did not inhibit the growth of the LAB population. The yeast inhibited the growth of coliforms during fermentation, with a higher inoculum suppressing the population earlier at 24 h (IN5 and IN7) than at 48 h (IN3). This highlights the ability of yeasts to control the coliforms, which can be sources of pathogens in the early stages of fermentation.

In the current study, yeast and LAB interactions remained active until the end of fermentation, as evidenced by the growing microbial population. This interaction accelerated fermentation and altered protein functional properties. The *P. kudriavzevii* inoculum (IN5 and IN3) are recommended to inhibit the coliform population during sorghum fermentation at 24 or 48 h, aligning with the end of fermentation for the latter. Furthermore, the inocula IN3 and IN5 enhanced nutritional quality (IVPD) and protein‐related functionality (WAC, NSI, and FAN), without compromising the availability of soluble nitrogen. Although IN7 achieved results similar to those of IN3 and IN5, the higher microbial population (LAB, yeasts, and TAC) can compromise the product′s microbial stability. Future studies on the probiotic properties of the yeast used in making the product are envisaged to develop a functional food product suitable for Southern African consumers.

## Funding

This study was supported by the Mastercard Foundation Scholars Program, National Research Foundation, South Africa, 129767; and Department of Science and Technology‐NRF CoE in Food Security, 91490.

## Ethics Statement

The project has ethic clearance NAS011/2021 issued by the University of Pretoria, South Africa.

## Conflicts of Interest

The authors declare no conflicts of interest.

## Data Availability

The data that support the findings of this study are available from the corresponding author upon reasonable request.
